# Prognostic impact of tumor size on cancer-specific survival for postoperative WHO grade II oligodendroglioma: a SEER-based study

**DOI:** 10.3389/fsurg.2025.1455567

**Published:** 2025-02-03

**Authors:** Qin Lu, Yongyan Wu, Yonglin Xie, Shuxu Yang, Hongchuan Jin

**Affiliations:** 1 Department of Neurosurgery, Sir Run Run Shaw Hospital, Zhejiang University, School of Medicine, Hangzhou, Zhejiang, China; 2 Department of Emergency, Sir Run Run Shaw Hospital, Zhejiang University, School of Medicine, Hangzhou, Zhejiang, China; 3 Laboratory of Cancer Biology, Key Lab of Biotherapy, Sir Run Run Shaw Hospital, Zhejiang University, School of Medicine, Hangzhou, Zhejiang, China

**Keywords:** WHO grade II oligodendroglioma, SEER database, prognosis, survival analysis, tumor size

## Abstract

**Background:**

WHO grade II oligodendroglioma (OG/II) is a rare primary brain tumor with various outcomes. Our study aims to investigate prognostic factors for postoperative OG/II patients and then evaluate the instructional value of tumor size.

**Methods:**

We retrospectively studied the cases from the Surveillance, Epidemiology, and End Results (SEER) database. Univariate and multivariate Cox analyses and Kaplan-Meier survival curves were used to identify and assess prognostic factors. The optimal cut-off value of tumor size was determined by X-tile analysis and verified by multivariate analyses. Subsequently, Subgroup analyses were performed based on tumor size.

**Result:**

646 OG/II patients were enrolled in our study. Multivariate Cox analyses revealed that age > 60 (HR 3.52), male (HR 1.48), total resection (HR 0.38), and tumor size (HR 2.04) were independent factors in predicting cancer-specific survival (CCS). The optimal cut-off value for tumor size was 60 mm. Patients with tumor size greater than 60 mm, age > 60 (HR 3.82), and radiation (HR 1.58) were associated with worse CSS, while total resection (HR 0.35) was associated with better CSS. Lastly, a tumor size-based nomogram was established objectively and accurately.

**Conclusion:**

Our study identified four crucial prognostic factors related to CSS in postoperative OG/II patients: age, sex, the extent of resection, and tumor size. A tumor size of 60 mm was an optimal cut-off point for dividing patients into low and high-risk groups. Patients in the low-risk group may not benefit from extended resection and radiation. Tumor size can be a valuable factor for making therapeutic schedules.

## Introduction

Oligodendroglioma is a rare primary brain tumor that is challenging to cure, and it originates from oligodendrocytes or glial precursor cells (1), constituting 2%–5% of all central nervous system (CNS) tumors (2). According to the World Health Organization (WHO) classification guidelines, oligodendrogliomas are characterized by the presence of an IDH mutation and 1p/19q codeletion (3). Based on tumor cells' integrated histological and molecular features, oligodendrogliomas can be divided into well-differentiated WHO grade II and anaplastic WHO grade III categories (4).

Given their infrequent occurrence, grade II and III oligodendrogliomas are often combined into an entity or grouped with astrocytic tumors during clinical investigations (5). Limited studies have identified the clinical and biological prognostic factors to predict the outcome of WHO II oligodendrogliomas (OG/II) patients (6), which exhibit lower malignancy compared to anaplastic oligodendrogliomas (AOG). Meanwhile, the therapeutic approaches for OG/II and AOG are usually different. For AOG, the recommended treatment protocol includes maximal safe surgical resection followed by radiation and chemotherapy (3, 7). However, the therapy for OG/II remains controversial. According to the 2022 National Comprehensive Cancer Network (NCCN) guidelines, OG/II is divided into low- and high-risk groups depending on the age and extent of resection (8). Nonetheless, as one of the important clinical features, tumor size was not considered a risk factor above.

In this study, a retrospective analysis was conducted utilizing data from the Surveillance, Epidemiology, and End Results (SEER) database. Clinical characteristics and independent prognostic factors were analyzed in OG/II patients. Furthermore, an optimal cut-off value for tumor size was established to identify patients with a poor prognosis, leading to the stratification of patients into two subgroups based on tumor size. Subsequent analyses were performed to elucidate the impacts of tumor size on the prognosis of OG/II patients, which is instructive for therapeutic strategies, such as the extent of resection, radiation, and chemotherapy.

## Material and methods

### Data collection

Data were collected from the SEER database (version 8.4.1), and patients diagnosed with WHO-II grade oligodendroglioma (ICD-O-3 histologic code 9450) from 1975 to 2020 were chosen in this study. Our data included age, sex, race, primary site, tumor size, surgery, radiation, survival time, and vital status.

**Figure 1:**
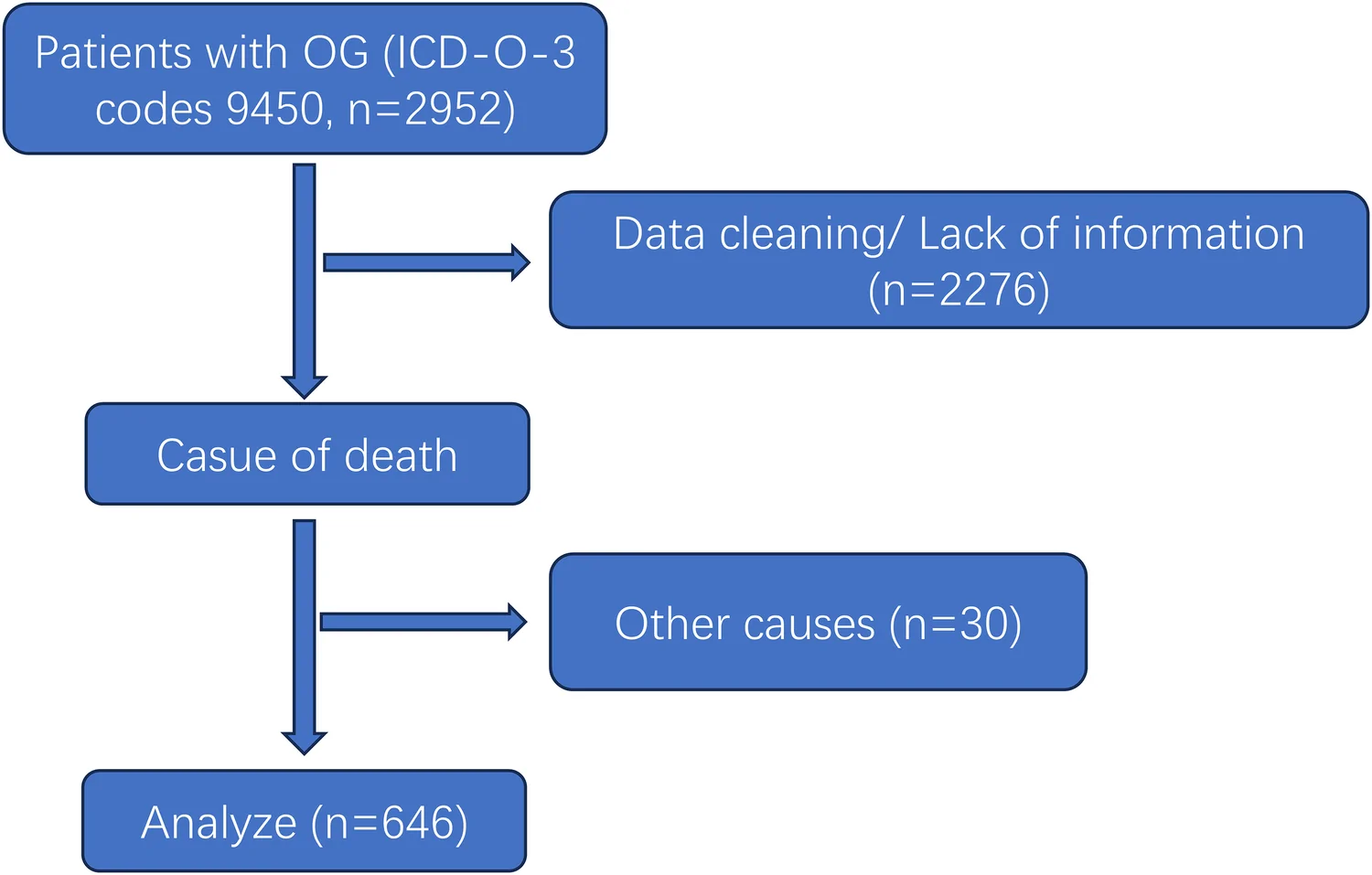
Flowchart of OG/II patient selection.

An extraction workflow of cases is presented in Figure 1. A total of 2,952 cases were found in the SEER database, and we cleaned data as follows (1): cases that lacked surgical information or were not surgical were excluded. The surgical information was classified into biopsy, subtotal resection, total resection, and extended resection. (2) Tumor size was collected according to the terms “CS Tumor Size”, and cases with unknown tumor sizes were cleaned, including the CS tumor size codes 999, 990, and 000. (2) cases that died due to other diseases were omitted. The endpoint of this study was cancer-specific survival. Finally, we confirmed the grades of selected cases, and all cases were moderately differentiated, meaning grade II. All data were collected and analyzed by two independent researchers and verified by the third one.

### Statistical analysis

All statistical analyses were performed with R (version 4.1.3), and the following R packages were used: “rms,” “foreign,” “survival,” “ggplot2,” “survminer,” and “forestplot.” Statistical significance was set at *P* < 0.05. Univariable and multivariable regression analyses were performed in all patients using a Cox proportional hazard model, and the results were presented as hazard ratios (HR) with corresponding 95% confidence intervals (CIs). Variables considered clinically relevant or showed a univariate relationship with outcome were entered into a multivariate Cox regression model. Survival was assessed using Kaplan-Meier models, and statistical significance was determined using the log-rank test. A prognostic nomogram was constructed by R to predict the survival of patients, and calibration curves were formulated to evaluate the judgment ability of the nomogram.

## Results

### Patients characteristics

A total of 646 postoperative WHO-II grade oligodendroglioma (OG/II) patients were collected in this study, with the diagnostic years ranging from 2004 to 2020 (Figure 1). The clinical characteristics of selected patients are listed in Table 1. Among them, 47.52% of patients were <40 years old, 87.15% were white, and 56.97% were male. Concerning tumor location, the majority of OG/II cases (62.23%) were situated in the frontal lobe.

**Table 1:** Characteristics of included patients.

Characteristic	Number of patients	Rate
Age, years		
0–39	307	47.52%
40–59	269	41.64%
>60	70	10.84%
Race		
White	563	87.15%
Black	29	4.49%
Others	54	8.36%
Sex		
Female	278	43.03%
Male	368	56.97%
Location		
Frontal lobe	402	62.23%
Temporal lobe	107	16.55%
Parietal lobe	72	11.15%
Occipital lobe	10	1.55%
Overlapping lesion	41	6.35%
Cerebrum	14	2.17%
Tumor size		
≤60 mm	527	81.58%
>60 mm	119	18.42%
Surgery		
Biopsy	133	20.59%
Subtotal resection	135	20.90%
Total resection	197	30.50%
Extended resection	181	28.01%
Radiation		
No	407	63.00%
Yes	239	37.00%
Total	646	

### Identification and validation of cut-off value for tumor size

**Figure 2:**
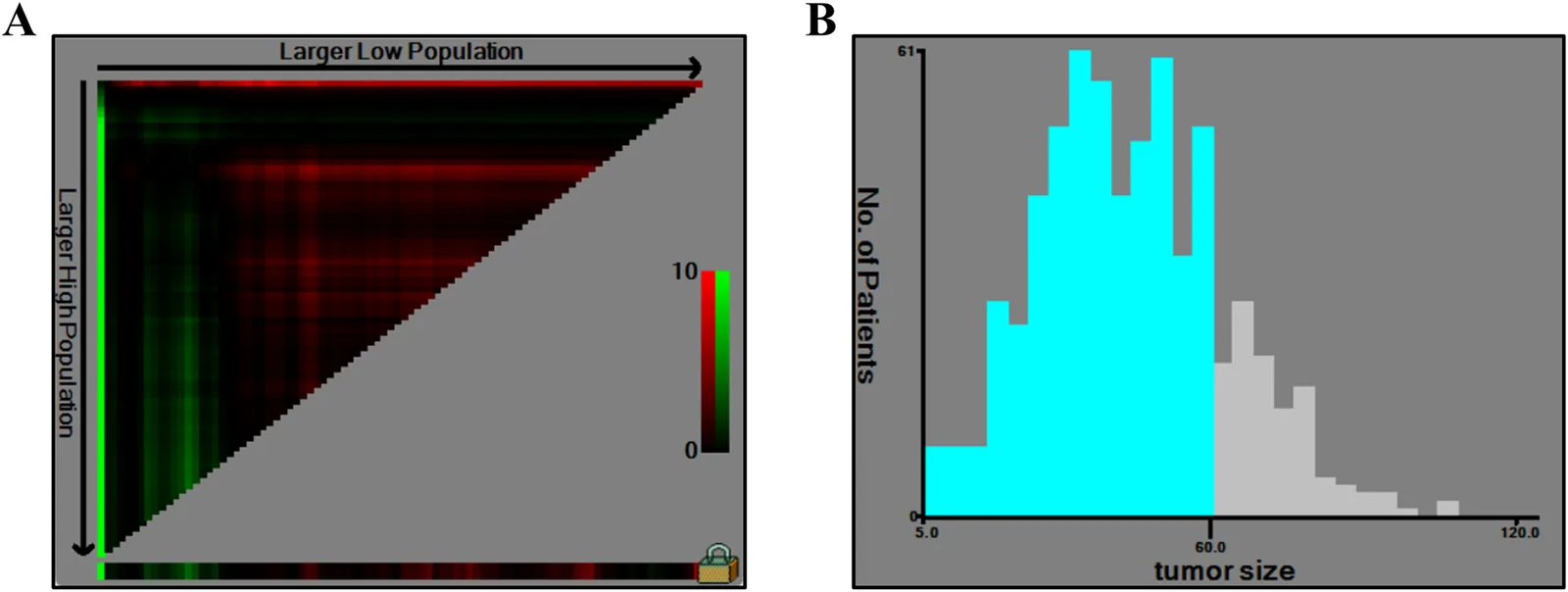
X-tile analysis of tumor size. **(A)** The graph shows that the optimal cutoff value was determined by X-tile software. **(B)** A histogram shows the distribution of tumor size values among patients.

The X-tile software was applied to analyze the optimal tumor size cut-off based on survival information. The cut-off value was identified by maximizing the chi-square score and minimizing the *P* value. As shown in Figure 2, 60 mm was identified as a suitable cut-off value. Furthermore, we also validated the cut-off value in increments of 5 mm using both univariate and multivariate Cox regression analyses (Table 2). Multivariate analysis showed that the *P* values of cut-off values from 30 to 75 mm were significant, and the tumor size cut-off at 60 mm had a high HR (2.035, 95% CI 1.390–2.981) for CSS. Although the largest HR was a cut-off value of 20 mm, univariate analysis has no significance. Thus, 60 mm was confirmed to be the optimal cut-off value for tumor size.

**Table 2:** Univariate and multivariate Cox analysis of different tumor size cutoffs in OG/II patients.

Tumor size	Number of patients	Univariate analysis			Multivariate analysis		
		HR (95% CI)	Forest plot	*P*	HR (95% CI)	Forest plot	*P*
≤10/>10 mm	14/632	0.845 (0.311–2.294)	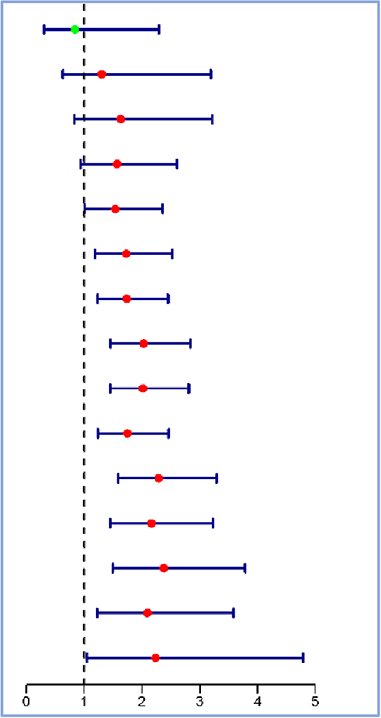	0.742	0.974 (0.349–2.719)	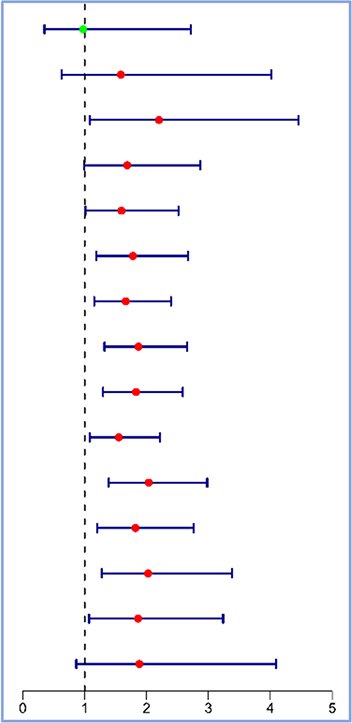	0.960
≤15/>15 mm	25/621	1.305 (0.633–3.194)		0.560	1.585 (0.625–4.018)		0.332
≤20/>20 mm	55/591	1.633 (0.830–3.212)		0.155	2.200 (1.086–4.457)		0.029
≤25/>25 mm	101/545	1.569 (0.945–2.607)		0.082	1.689 (0.992–2.871)		0.054
≤30/>30 mm	148/498	1.541 (1.006–2.360)		0.047	1.560 (1.012–2.516)		0.044
≤35/>35 mm	218/428	1.731 (1.185–2.528)		0.005	1.781 (1.188–2.670)		0.005
≤40/>40 mm	291/355	1.738 (1.232–2.453)		0.002	1.663 (1.155–2.397)		0.006
≤45/>45 mm	352/294	2.031 (1.453–2.839)		<0.001	1.871 (1.318–2.656)		<0.001
≤50/>50 mm	425/221	2.018 (1.450–2.808)		<0.001	1.830 (1.296–2.584)		<0.001
≤55/>55 mm	469/177	1.748 (1.242–2.461)		0.001	1.551 (1.086–2.215)		0.016
≤60/>60 mm	527/119	2.289 (1.590–3.295)		<0.001	2.035 (1.390–2.981)		<0.001
≤65/>65 mm	561/85	2.165 (1.453–3.227)		<0.001	1.819 (1.199–2.759)		0.005
≤70/>70 mm	592/54	2.379 (1.494–3.788)		<0.001	2.025 (1.274–3.378)		0.003
≤75/>75 mm	605/41	2.095 (1.226–3.582)		0.007	1.863 (1.072–3.237)		0.027
≤80/>80 mm	627/19	2.236 (1.044–4.789)		0.038	1.882(0.864–4.096)		0.111

### Factors associated with cause-specific survival

Univariate and multivariate COX analyses evaluated the associations of characteristics in Table 1 with cause-specific survival (CSS). Univariate analysis indicated that age (>60 years vs. <40 years, HR 3.82, 95% CI 2.45–5.97, *P* < 0.01), sex (male vs. female, HR 1.45, 95% CI 1.03–2.04, *P* = 0.031), tumor size (>60 mm vs. ≤60 mm, HR 2.29, 95% CI 1.59–3.30, *P* < 0.01), surgery (total resection vs. biopsy, HR 0.36, 95% CI 0.21–0.64, and extended resection vs. biopsy, HR 0.62, 95% CI 0.42–0.82, *P* = 0.019), and radiation (no vs. yes, HR 1.70, 95% CI 1.22–2.38, *P* = 0.002) were significantly related to CSS of OG/II patients. Meanwhile, a similar result was found in multivariate COX analyses, except for radiation (Table 3). The result suggested that age, sex, tumor size, and surgery were the key prognostic factors for OG/II patients.

**Table 3:** Univariate and multivariate analyses of cause-specific survival.

Variable	Univariate		Multivariate	
	*P* value	HR (95% CI)	*P* value	HR (95% CI)
Age				
0–39	Reference	Reference	Reference	Reference
40–59	0.148	1.32 (0.91–1.91)	0.569	1.12 (0.76–1.65)
>60	0.000	3.82 (2.45–5.97)	0.000	3.52 (2.23–5.58)
Race				
White	Reference	Reference	–	–
Black	0.296	1.47 (0.72–3.00)	–	–
Others	0.947	1.00 (0.53–1.82)	–	–
Sex				
Female	Reference	Reference	Reference	Reference
Male	0.033	1.45 (1.03–2.04)	0.027	1.48 (1.05–2.10)
Location				
Frontal lobe	Reference	Reference	Reference	Reference
Temporal lobe	0.295	1.26 (0.82–1.94)	0.472	1.17 (0.76–1.81)
Parietal lobe	0.523	1.20 (0.69–2.08)	0.607	1.16 (0.66–2.05)
Occipital lobe	0.09	2.33 (0.85–6.36)	0.252	1.83 (0.65–5.15)
Overlapping lesion	0.06	1.81 (0.96–3.40)	0.394	1.33 (0.69–2.53)
Cerebrum	0.234	1.84 (0.67–5.035)	0.335	1.66 (0.59–4.61)
Tumor size				
≤60 mm	Reference	Reference	Reference	Reference
>60 mm	0.000	2.29 (1.59–3.30)	0.000	2.04 (1.39–2.99)
Surgery				
Biopsy	Reference	Reference	Reference	Reference
Subtotal resection	0.054	0.59 (0.34–1.01)	0.015	0.51 (0.29–0.88)
Total resection	0.000	0.36 (0.21–0.64)	0.001	0.38 (0.22–0.67)
Extended resection	0.019	0.62 (0.42–0.92)	0.066	0.67 (0.45–1.03)
Radiation				
No	Reference	Reference	Reference	Reference
Yes	0.002	1.70 (1.22–2.38)	0.119	1.32 (0.93–1.89)

**Figure 3:**
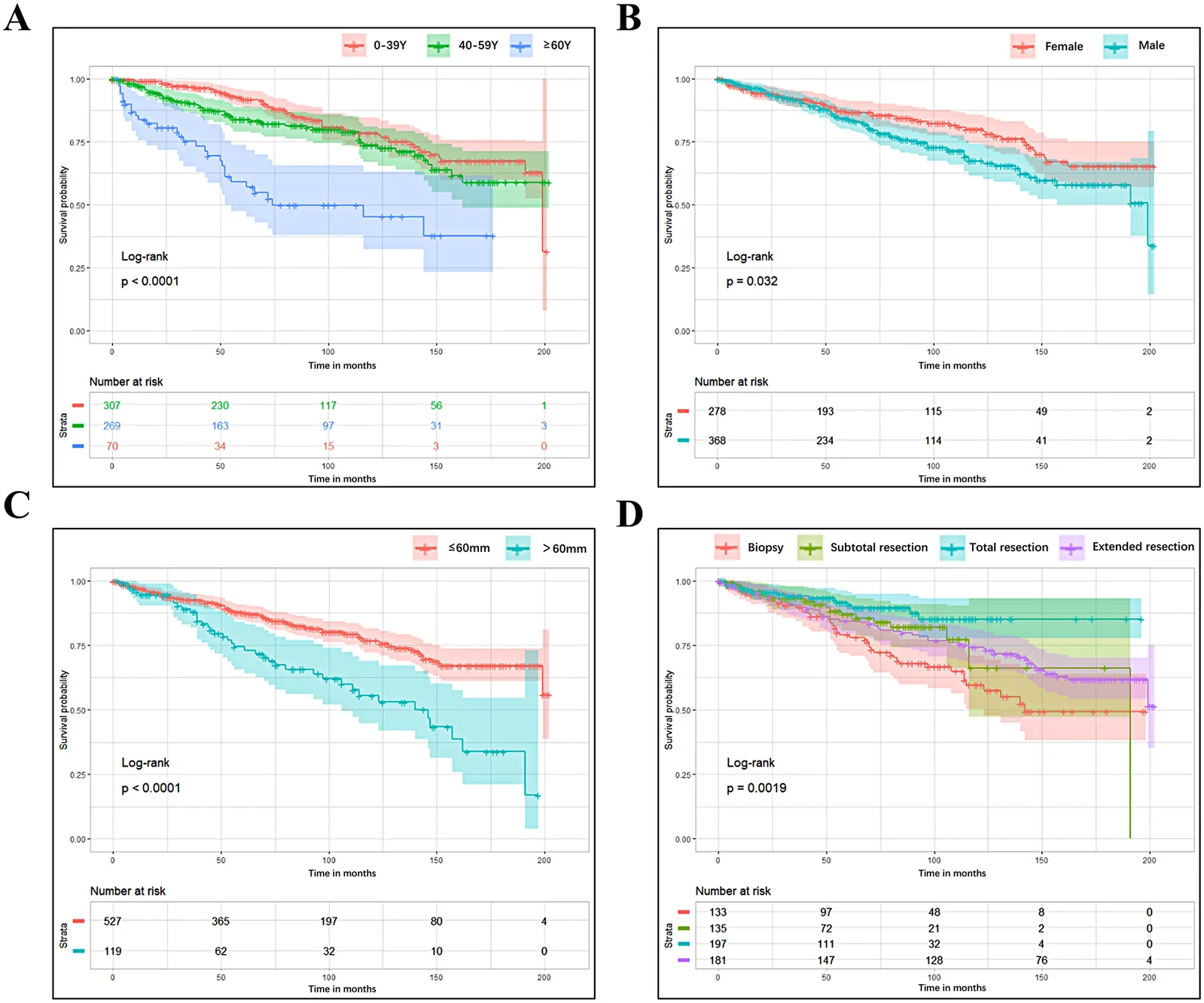
The Kaplan-Meier curves of CSS for OG/II patients. **(A)** age; **(B)** sex; **(C)** tumor size; **(D)** the extent of resection.

Kaplan-Meier curves were subsequently conducted on these four factors, and the result indicated that patients older than 60 years (Figure 3A) or males (Figure 3B) lived shorter. In contrast, those with tumor sizes smaller than 60 mm (Figure 3C) or operated with total resection (Figure 3D) had a longer survival time.

### Impacts of tumor size on treatment outcomes

Based on the tumor size, the OG/II patients were categorized into a low-risk group (*n* = 527) and a high-risk group (*n* = 119). The characteristics of patients in these groups are detailed in Table 4. Univariate and multivariate COX analyses were conducted (Table 5). In the low-risk group, multivariate analyses indicated that an age greater than 60 years (HR 4.05) was associated with poor CSS. In contrast, surgery, especially for total resection (HR 0.35), was related to improved CSS. In the high-risk group, only age and sex were considered independent predictors for CSS. Furthermore, an unfavorable role of radiation (HR 1.58, *P* = 0.033) was found in the low-risk group, and a favorable tendency of radiation (HR 0.98) was observed in the high-risk group. However, the tendency lacked statistical significance.

**Table 4:** Characteristics of patients in two groups.

Characteristic	Low risk		High risk	
	Number of patients	Rate	Number of patients	Rate
Age, years				
0–39	257	48.77%	50	42.02%
40–59	214	40.61%	55	46.22%
>60	56	10.62%	14	11.76%
Race				
White	461	87.48%	102	85.72%
Black	26	4.93%	3	2.52%
Others	40	7.59%	14	11.76%
Sex				
Female	237	44.97%	41	34.45%
Male	290	55.03%	78	65.55%
Location				
Frontal lobe	330	62.62%	72	60.51%
Temporal lobe	90	17.08%	17	14.29%
Parietal lobe	62	11.76%	10	8.40%
Occipital lobe	9	1.71%	1	0.84%
Overlapping lesion	27	5.12%	14	11.76%
Cerebrum	9	1.71%	5	4.20%
Surgery				
Biopsy	111	21.06%	22	18.49%
Subtotal resection	99	18.79%	36	30.25%
Total resection	169	32.07%	28	23.53%
Extended resection	148	28.08%	33	27.73%
Radiation				
No	352	66.79%	55	46.22%
Yes	175	33.21%	64	53.78%
Total	527		119	

**Table 5:** Univariate and multivariate analyses in two groups.

Variable	Low risk (527)				High risk (119)			
	Univariate		Multivariate		Univariate		Multivariate	
	*P* value	HR (95% CI)	*P* value	HR (95% CI)	*P* value	HR (95% CI)	*P* value	HR (95% CI)
Age								
0–39	Reference	Reference	Reference	Reference	Reference	Reference	Reference	Reference
40–59	0.201	1.34 (0.86–2.08)	0.447	1.19 (0.76–1.88)	0.951	1.02 (0.51–2.05)	0.900	1.01 (0.46–2.21)
>60	0.000	4.05 (2.40–6.83)	0.000	3.82 (2.20–6.65)	0.030	2.60 (1.10–6.13)	0.003	4.67 (1.67–13.08)
Race								
White	Reference	Reference	–	–	Reference	Reference	Reference	Reference
Black	0.472	1.35 (0.59–3.10)	–	–	0.083	3.61 (0.84–15.47)	0.334	2.33 (0.42–12.89)
Others	0.652	0.83 (0.36–1.89)	–	–	0.814	1.12 (0.43–2.89)	0.645	1.26 (0.47–3.43)
Sex								
Female	Reference	Reference	Reference	Reference	Reference	Reference	Reference	Reference
Male	0.138	1.35 (0.91–2.01)	0.316	1.23 (0.82–1.86)	0.224	1.51 (0.78–2.93)	0.027	2.42 (1.11–5.31)
Location								
Frontal lobe	Reference	Reference	Reference	Reference	Reference	Reference	Reference	Reference
Temporal lobe	0.118	1.48 (0.91–2.41)	0.239	1.35 (0.82–2.21)	0.862	0.92 (0.35–2.40)	0.737	0.84 (0.31–2.31)
Parietal lobe	0.363	1.34 (0.71–2.50)	0.734	1.11 (0.59–2.13)	0.852	1.12 (0.33–3.76)	0.452	1.65 (0.45–6.11)
Occipital lobe	0.034	3.01 (1.09–8.31)	0.182	2.05 (0.72–5.85)	0.997	0.00 (0.00-Inf)	0.997	0.00 (0.00-Inf)
Overlapping lesion	0.383	1.50 (0.60–3.76)	0.867	1.08 (0.43–2.74)	0.372	1.51 (0.61–3.74)	0.267	1.83 (0.63–5.30)
Cerebrum	0.168	2.27 (0.71–7.26)	0.127	2.52 (0.77–8.23)	0.997	1.00 (0.13–7.58)	0.972	0.96 (0.12–7.79)
Surgery								
Biopsy	Reference	Reference	Reference	Reference	Reference	Reference	Reference	Reference
Subtotal resection	0.042	0.50 (0.26–0.98)	0.012	0.42 (0.22–0.83)	0.570	0.75 (0.28–1.99)	0.642	0.78 (0.27–2.23)
Total resection	0.001	0.33 (0.18–0.64)	0.001	0.35 (0.18–0.67)	0.323	0.55 (0.17–1.79)	0.418	0.60 (0.35–2.08)
Extended resection	0.010	0.54 (0.34–0.87)	0.052	0.62 (0.38–1.00)	0.924	0.96 (0.44–2.12)	0.709	0.85 (0.35–2.04)
Radiation								
No	Reference	Reference	Reference	Reference	Reference	Reference	Reference	Reference
Yes	0.004	1.80 (1.21–2.69)	0.033	1.58 (1.04–2.39)	0.713	0.89 (0.47–1.67)	0.975	0.98 (0.48–2.04)

### Construction of nomogram for the low-risk group

**Figure 4:**
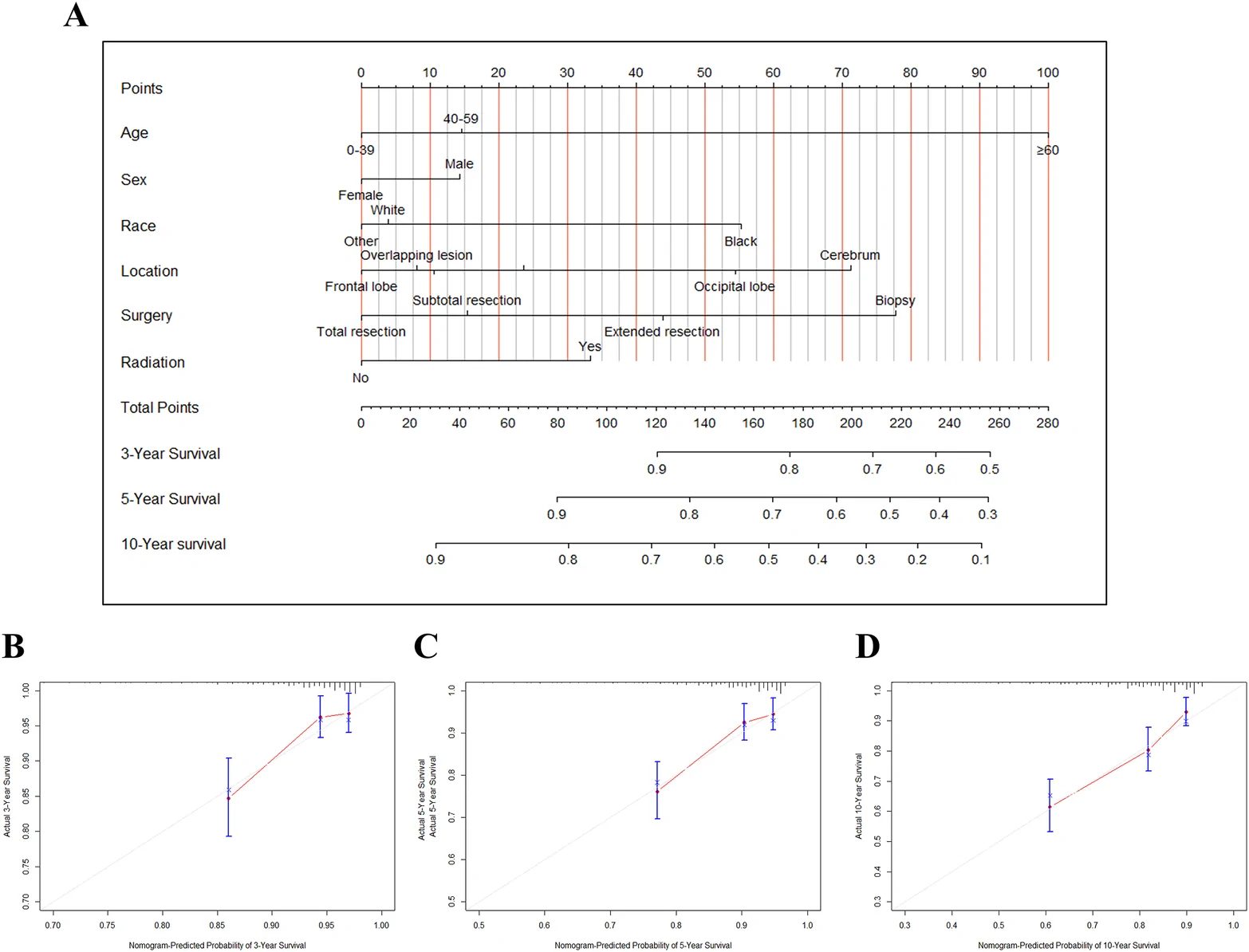
The nomogram and calibration plots for predicting survival of OG/II patients with tumor size ≤ 60 mm. **(A)** The nomogram for predicting 3-,5-, and 10-year survival. Calibration plots for 3-year **(B)**, 5-year **(C)**, and 10-year **(D)** survival prediction.

A nomogram was constructed to predict the survival of postoperative OG/II patients with a tumor size less than 60 mm. As shown in Figure 4A, each patient's corresponding survival probability could be obtained by summing each predictor's total scores. For example, a 60-year-old male white patient was diagnosed with an oligodendroglioma in the occipital lobe, and he underwent a subtotal resection of the tumor without additional radiation. According to the nomogram, the predicted 3-,5-, and 10-year survival rates are about 81%, 70%, and 50% respectively. Furthermore, The calibration curves of the 3-, 5-, and 10-year survival rates showed good agreement between the nomogram predictions and actual observations (Figures 4B–D).

## Discussion

The clinic's prediction of oligodendroglioma outcomes remains challenging due to its rare incidence. Specifically, accurate and effective prognostication of OG/II, a subtype of oligodendroglioma, is critical for personalized therapeutic approaches and may present additional difficulties. Thus, we conducted a retrospective analysis on SEER, a database that offers an opportunity to investigate rare diseases.

**Figure 5:**
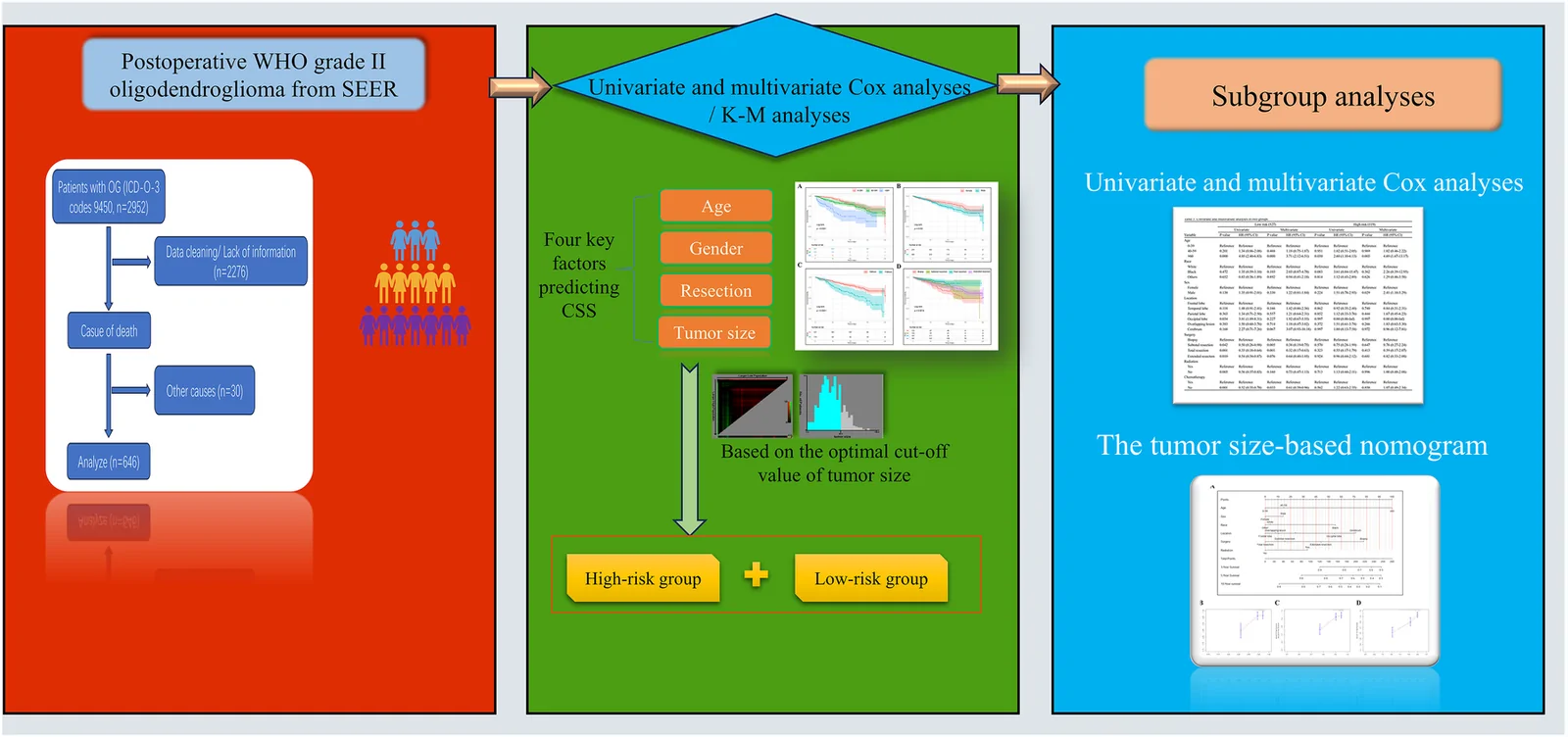
Schematic of study.

Our study collected and analyzed 646 postoperative OG/II cases from SEER. Four key prognostic factors related to CCS in postoperative OG/II patients were identified, including age, sex, excision extension, and tumor size. OG/II can be classified into low- and high-risk groups based on tumor size. Total resection is recommended in the low-risk group, while extended resection and radiation may not be beneficial. Tumor size can be a valuable factor for predicting prognosis and making therapeutic schedules, and a nomogram was established (Figure 5).

Nearly half of our cases were younger than 40 years, with the incidence peak at 30–39 years. A similar result, 36–40 years, was reported in a study based on the Central Brain Tumor Registry of the United States (CBTRUS) from 2000 to 2013 (9). Our analysis indicated that age is an important factor affecting the prognosis of OG/II patients, and patients over 60 years had worse survival when compared to those younger than 40 years. Age is also considered an essential prognostic factor in other types of glioma (10). Research has demonstrated that glioma is more aggressive in elderly patients (11). Older men usually do not recommend surgery and adjuvant treatment (12). Thus, younger patients are more likely to have a better prognosis. Elderly patients are more prone to have comorbidities, which makes them more susceptible to death from factors other than tumors. During the process of organizing our data, we found that patients over the age of 60 have a 23.3% chance of dying from non-tumor-related causes. In contrast, in patients under the age of 60, the rate of non-tumor-related mortality is only 7.5%. Thus, we chose to focus on cancer-specific survival (CCS) to mitigate the potential impact of comorbidities.

Sex is another independent prognostic factor in our study, as shown by univariate and multivariate analysis. In the present study, the male gender was related to high tumor-specific mortality compared to females. As reported, sex differences have been well-identified in many brain tumors, such as glioblastoma. Glioma patients usually present a greater tumor incidence and worse outcomes in males, which may be caused by differences in pathophysiological mechanisms such as hormonal influences, metabolic pathways, immune responses, and molecular changes (13). Understanding the role of gender in OG/II may help to create a sex-specific therapy to improve the survival of patients.

Maximal safe resection of the tumor is the first and most recommended therapy for glioma. However, for OG/II, the influence of tumor resection on the prognosis seems very mild (14). The extent of surgical tumor resection remains controversial. Shawn L. et al. conducted a retrospective study on a multicenter and multinational cohort of 757 diffuse low-grade glioma (LGG) patients. Their result indicated that the extent of surgical tumor resection beginning at 75% improves over survival while beginning at 80% improves progression-free survival of LLG patients (15). However, this result relied on the combined analysis of oligodendrogliomas and astrocytomas. Connor J. et al. reported that a greater extent of surgical tumor resection is associated with improved survival in oligodendrogliomas (16), which include OG/II and AOG. Our study focused on OG/II and found that extended resection of tumors can not benefit more than subtotal resection. And patients with total resection had better cause-specific survival. Thus, we recommended a total tumor resection for OG/II.

Tumor size is the maximum diameter of the tumor and has been proven to be a critical prognostic factor for many tumors, such as lung cancer (17), uterine sarcoma (18), and hepatoma (19). In neuroblastoma, Wang et al. identified a cut-off value of 4 cm for tumor size and suggested that tumor size >4 cm might predict poor prognosis (20). Lin et al. reported a tumor size of 59 mm as a critical cut-off value for low-grade supratentorial glioma, and they suggest that a tumor size >59 mm represents a high risk and indicates a worsened outcome (21). However, few studies investigate the value of tumor size for OG/II patients. We identified and verified a cut-off value of 60 mm for OG/II, which indicated that tumor size >60 mm was a high risk for postoperative OG/II patient survival.

Therapeutic schedules for OG/II patients remain controversial and should mainly focus on prognostic factors. ASCO-SNO Guidelines suggest surgical resection accompanied by radiation and chemotherapy for oligodendroglioma, including OG/II and AOG, but the strength of the recommendation is weak (3). A risk classification system was introduced in 2022 NCCN guidelines, and OG/II is divided into low- and high-risk groups relying on the age and extent of resection (8). However, this classification did not include the tumor size, an essential prognostic factor in our analysis. Thus, we further divided the patients into a low-risk group (tumor size ≤60 mm) and a high-risk group (tumor size >60 mm). The result of univariate and multivariate analyses suggested subtotal or total resection in low-risk patients. However, extended resection and radiation were not recommended in patients with tumor sizes smaller than 60 mm. The result agrees with the biological behavior of OG/II, which is a benign tendency with relatively slow tumor growth (15, 22). When making a therapeutic schedule, it is important to consider the benefits and potential damage of treatment such as surgery, radiation, and chemotherapy (23). In patients with tumor sizes larger than 60 mm, we found beneficial roles in CSS of radiation with HR < 1, which suggested an adjuvant treatment. However, the results were not statistically significant with *P* values > 0.05, possibly due to the small sample size. More studies are needed to clarify the results.

There are several limitations to our study. Firstly, this is a retrospective study performed on SEER data, and some potential biases can not be avoided, such as incomplete data and misclassification of variables. We are eager for more studies to confirm our idea, especially for randomized controlled trials. Secondly, many variables do not exist or are incomplete in the SEER database. Still, they are closely related to survival, such as the details of chemotherapy, duration of symptoms, etc. Thirdly, the cases in the high-risk group are not big enough, and we can not get a useful and significant result in this group. Lastly, we established a nomogram to predict the survival of OG/II patients with tumor sizes less than 60 mm. An external validation cohort may be needed to assess the applicability in the patients. Other variables, like background disease, need to be considered in the nomogram in future studies.

## Conclusion

In summary, our study identified four critical prognostic factors in postoperative WHO-II grade oligodendroglioma: age, sex, the extent of resection, and tumor size. We established a cut-off value of 60 mm for tumor size, which allowed us to classify OG/II into low- and high-risk groups. Further analysis indicates that total resection is advantageous for patients with tumor sizes less than 60 mm, and subtotal resection also appears to be favorable. However, extended resection and radiation therapy may not confer additional benefits on these patients. Furthermore, A nomogram established in the present study could predict the prognosis for OG/II patients with tumor size less than 60 mm objectively and accurately. However, more studies are required to confirm our conclusion.

## Data Availability

Publicly available datasets were analyzed in this study. This data can be found here: Our original data are obtained from a public database(SEER), and the analyzed data are available from the corresponding author on reasonable request.
