# Correction: Prognostic impact of tumor size on cancer-specific survival for postoperative WHO grade II oligodendroglioma: a SEER-based study

**DOI:** 10.3389/fsurg.2026.1913452

**Published:** 2026-09-18

**Authors:** Qin Lu, Yongyan Wu, Yonglin Xie, Shuxu Yang, Hongchuan Jin

**Affiliations:** 1 Department of Neurosurgery, Sir Run Run Shaw Hospital, Zhejiang University, School of Medicine, Hangzhou, Zhejiang, China; 2 Department of Emergency, Sir Run Run Shaw Hospital, Zhejiang University, School of Medicine, Hangzhou, Zhejiang, China; 3 Laboratory of Cancer Biology, Key Lab of Biotherapy, Sir Run Run Shaw Hospital, Zhejiang University, School of Medicine, Hangzhou, Zhejiang, China

**Keywords:** WHO grade II oligodendroglioma, SEER database, prognosis, survival analysis, tumor size

There was a mistake in Figure 3 as published. Figure 3 shows survival data in days instead of in months. The corrected Figure 3 appears below.

There was a mistake in the caption of Figure 3 as published. There was a typo in the caption and the word “resection” was misspelled as “recession”. The corrected caption of Figure 3 appears below.

**Figure 3:**
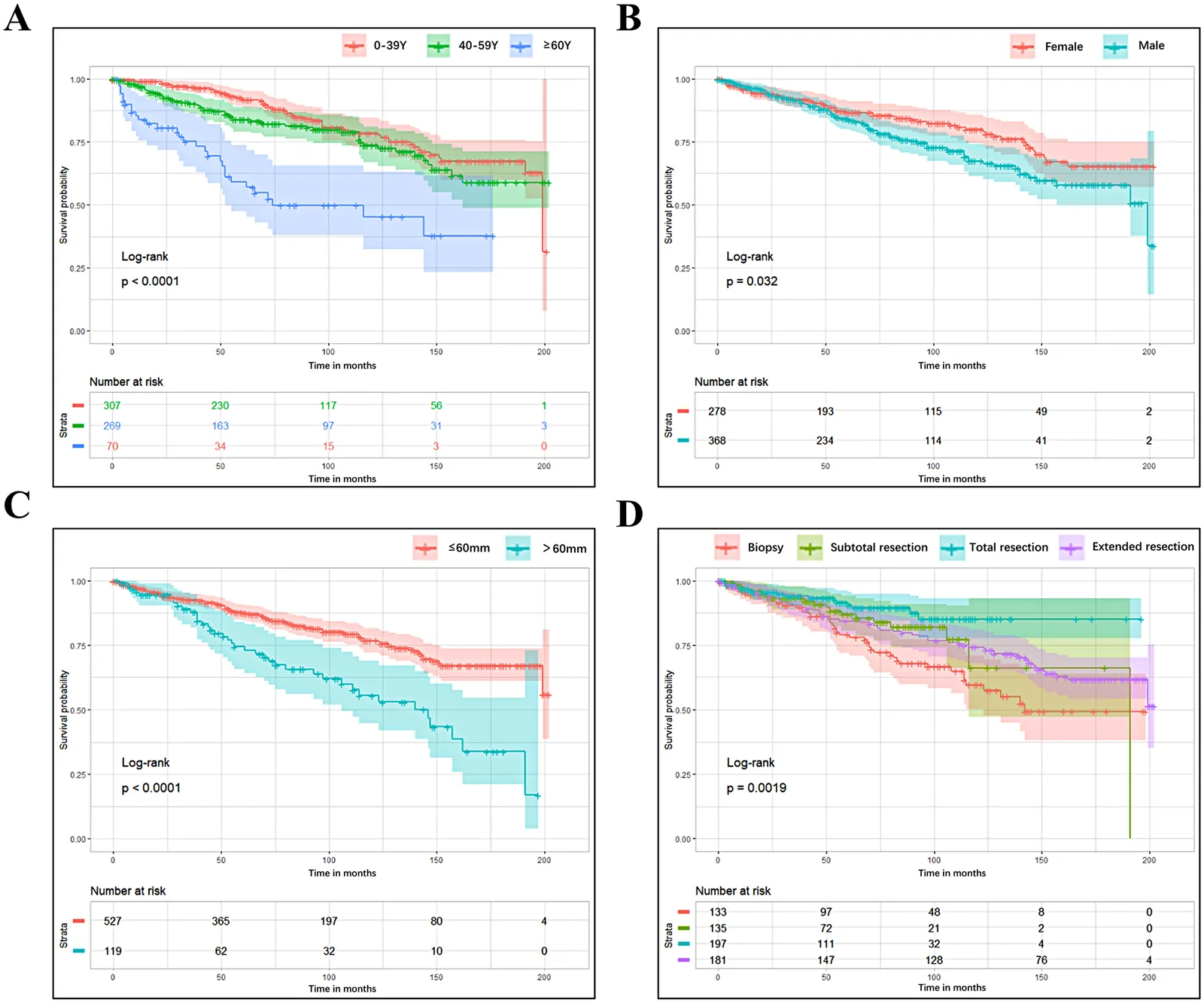


“Figure 3. The Kaplan–Meier curves of CSS for OG/II patients. (A) age; (B) sex; (C) tumor size; (D) the extent of resection.”

In the Abstract, it was incorrectly written as “Patients with tumor size less than 60 mm, age > 60 (HR 3.82), and radiation (HR 1.58) were associated with worse CSS,”. This has been corrected to read: “Patients with tumor size greater than 60 mm, age > 60 (HR 3.82), and radiation (HR 1.58) were associated with worse CSS,”

The word “resection” was misspelled as “recession” in the Abstract, in the section Discussion (paragraph 3) and in the section Conclusion (paragraph 3). This has been corrected.

A correction has been made to the Abstract and the Results section where the final cohort size number was written as 676 instead of 646.

The original version of this article has been updated.

